# Evaluation of Post-Processing Time’s Influence on Biocompatibility of 3D-Printed Denture Base Resins

**DOI:** 10.3390/jfb17040188

**Published:** 2026-04-12

**Authors:** Miruna Dinescu, Vlad Gabriel Vasilescu, Lucian Toma Ciocan, Bianca Voicu-Balasea, Ana Maria Cristina Țâncu, Alexandra Ripszky, Florin Miculescu, Sabina-Ana Răuță, Alexia-Ecaterina Cârstea, Mihaela Pantea, Marina Imre

**Affiliations:** 1 Department of Prosthodontics, Faculty of Dentistry, “Carol Davila” University of Medicine and Pharmacy, 37 Dionisie Lupu Street, District 2, 020021 Bucharest, Romania; miruna.dinescu@drd.umfcd.ro (M.D.); anamaria.tancu@umfcd.ro (A.M.C.Ț.); mihaela.pantea@umfcd.ro (M.P.); marina.imre@umfcd.ro (M.I.); 2 Department of Dental Prostheses Technology, Faculty of Dentistry, “Carol Davila” University of Medicine and Pharmacy, 37 Dionisie Lupu Street, District 2, 020021 Bucharest, Romania; vlad.vasilescu@umfcd.ro (V.G.V.); sabina-ana.rauta@drd.umfcd.ro (S.-A.R.); alexia-ecaterina.carstea2023@stud.umfcd.ro (A.-E.C.); 3 Department of Biochemistry, Faculty of Dental Medicine, “Carol Davila” University of Medicine and Pharmacy, 020021 Bucharest, Romania; alexandra.ripszky@umfcd.ro; 4 Faculty of Material Science and Engineering, National University of Science and Technology Politehnica Bucharest, 011061 Bucharest, Romania; f_miculescu@yahoo.com

**Keywords:** post-processing, denture base resin, digital light processing, light curing, cytotoxicity

## Abstract

In the continuous development of additive technologies and light-sensitive resins, the biological performance of 3D-printed resins is strongly dependent on photopolymerization efficiency and post-processing conditions. This study evaluated the effect of post-curing duration on the cellular response to two denture base resins using direct contact and indirect eluate-based pathways. Human gingival fibroblasts were assessed through viability, membrane integrity, nitric oxide production, fluorescence live/dead staining, and caspase-3/7 activity. As a result of contact between the cells and the surface interface of the specimen disks, reduced metabolic activity was noticed compared with the control under direct exposure, indicating cellular stress. Extended polymerization has been demonstrated to improve metabolic activity and reduce apoptotic signals for the V-Print dentbase resin, whereas FotoDent Denture presented a less uniform response under the same parameters. Therefore, for evaluating the cytotoxicity of light-sensitive resins, it is not sufficient to assess only the saliva-soluble substances released from the resin, such as residual monomers, but also the 3D printing parameters.

## 1. Introduction

Removable complete dentures remain a fundamental treatment option for edentulous patients, providing rehabilitation of oral function, esthetics, and comfort. As a gold standard, denture bases have been fabricated from polymethyl methacrylate (PMMA) because of its acceptable biocompatibility, ease of processing, and affordability. Despite these advantages, the conventional technique has several drawbacks, such as polymerization shrinkage, porosity, and long-term susceptibility to fracture and microbial colonization, which may compromise the oral tissue [1,2]. In addition, traditional denture fabrication workflows are labor-consuming and sensitive to operator technique, motivating the dental market for more efficient manufacturing approaches [3].

As an alternative to pack-and-press or injection molding, the appearance of digital dentistry offers CAD-CAM, enabling denture fabrication through both subtractive and additive processes [4]. The subtractive or milling technique for pre-polymerized PMMA blocks provides denture bases with high dimensional accuracy and a high degree of monomer conversion [5]; however, this approach is limited by considerable material waste and the inherent design restrictions of prefabricated blocks [1,6]. In contrast, additive or 3D-printing manufacturing offers the possibility of producing complex, anatoform morphologies while reducing material consumption and production time [1]. Among additive techniques, vat photopolymerization methods such as stereolithography (SLA) and digital light processing (DLP) have become the most used for denture base fabrication due to their high resolution and accuracy [7,8,9,10]. In vat photopolymerization, denture bases are fabricated from liquid photosensitive resins that polymerize upon exposure to light of a specific wavelength. These resins typically consist of methacrylate-based monomers, photoinitiators, and various additives that together determine the curing behavior and final properties of the printed object [11]. The efficiency of the photopolymerization process, referring to the degree of monomer-to-polymer conversion, is influenced by multiple factors such as resin components, light wavelength and intensity, exposure duration, and subsequent post-curing conditions [1,12,13]. Following the initial polymerization phase during the 3D printing process, the denture base presents porous areas that retain uncured resin, which must be removed through post-processing steps. First is the cleaning protocol, most commonly involving immersion in isopropyl alcohol. The next step is post-polymerization, typically performed in a dedicated ultraviolet curing unit and often combined with controlled heating, to promote further cross-linking of the polymer network [4,11]. All these procedures aim to improve the mechanical properties, dimensional stability, and biocompatibility of the final prosthesis. Furthermore, variations in printing orientation, layer thickness, curing time, and temperature have been demonstrated to influence surface characteristics and biological behavior, including cytotoxic response [6,8,11,14]. For this reason, the International Organization for Standardization (ISO) recommended cytotoxicity protocols that include both direct contact tests and indirect methods. Direct exposure evaluates the influence of surface characteristics and residual components at the material–cell interface, whereas indirect tests assess the biological effect of substances released from the polymer into the surrounding medium [15]. Among the common in vitro tests that evaluate cellular responses are metabolic activity, membrane integrity, apoptosis, and inflammatory stress [16].

The MTT assay is widely used to evaluate cell viability and metabolism. While LDH assesses the integrity of the cell membrane, higher levels reflect membrane damage and necrosis processes. Nitric oxide, as a signaling molecule, is produced by nitric oxide synthase through redox reactions; elevated NO levels are linked to cell inflammation and apoptosis caused by cytotoxic effects. Caspase-3/7 activity is recognized as a marker of apoptosis, allowing differentiation between reversible metabolic inhibition and programmed cell death [6]. In addition, live/dead fluorescence staining provides morphological confirmation of cell survival and membrane integrity, complementing viability assays.

Evidence-based research indicates that 3D-printed dental resins generally meet biocompatibility requirements when manufacturers’ post-processing protocols are well followed [4,14,15,17]. Although denture bases can be fabricated using different manufacturing approaches, their clinical performance depends on the polymerization of resin materials into a stable polymer network [1,18]. Conventional heat-polymerized polymethyl methacrylate (PMMA) is produced through thermal activation of methyl methacrylate monomers, which generally results in a high degree of conversion and relatively low residual monomer content when appropriate curing cycles are applied [7,18]. In contrast, light-cured denture base resins, such as Fotodent or V-print dentbase, are composed of methacrylate oligomers, reactive monomers, and photoinitiators, which polymerize upon light exposure during layer-by-layer fabrication [19,20,21]. Because this rapid process may lead to incomplete polymerization within and between layers, post-processing steps, particularly cleaning and post-curing, are critical to enhance polymer conversion and reduce residual monomer release [1,3,6].

Regarding the information provided by FotoDent Denture (Dreve) and V-Print dentbase (VOCO) producers, there are differences in both chemical composition and the post-processing protocols recommended by the manufacturers [19]. FotoDent Denture is composed mainly of polyether and urethane-based methacrylate oligomers. The cleaning step is recommended to be performed with isopropyl alcohol, followed by high-intensity light curing, often performed under controlled atmospheric conditions for reducing oxygen inhibition and fostering polymer chain formation [19,20]. V-Print dentbase consists primarily of aliphatic urethane dimethacrylate combined with triethylene glycol dimethacrylate and is recommended to be post-processed using alcohol-based cleaning and UV-lightbox curing in a light chamber at defined wavelengths and exposure durations. Given these differences in resin chemistry and curing strategy, variations in post-curing duration may influence residual monomer release in a material-dependent manner [19,21]. Although both materials are intended for long-term use as denture bases, differences in resin composition and photoinitiator systems suggest that their responses to post-curing parameters may not be identical. Therefore, variations in polymerization time, even within the ranges recommended by manufacturers, may influence the final properties of these materials.

## 2. Purpose

The present study aims to evaluate the effect of different post-curing times on the biocompatibility of two different 3D-printed denture base resins. According to the manufacturer’s instructions, a post-processing time of 30 min has a good result on cell viability after exposure to the materials. However, monomer residuals were reported to be present after this period [22]. Therefore, we hypothesized that an extended post-processing time (60 min) would impact the cytotoxicity effect of the materials.

## 3. Materials and Methods

### 3.1. Materials

#### 3.1.1. 3D-Printed Denture Base Resins

Two different 3D-printed denture base resins have been studied: FotoDent Denture, pink-transparent (Dreve Dentamid, Unna, Germany) and V-Print dentbase (VOCO GmbH, Cuxhaven, Germany).

According to the manufacturer’s technical sheet, the product descriptions and recommendations are different and systematized in Table 1 [20,21]:

We decided to use the following notation for the resins: F for FotoDent Denture resin and V for V-Print dentbase resin.

#### 3.1.2. Sample Preparation

**Figure 1:**
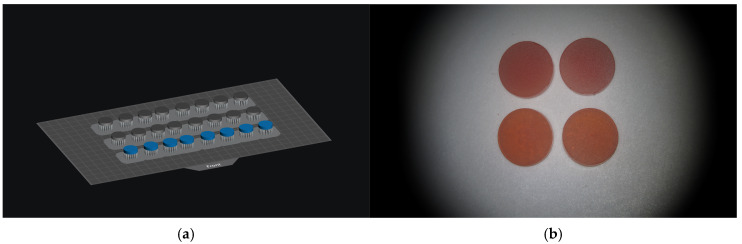
(**a**) Digital visualization of the disk-shaped specimens in the slicing software (Meshmixer v3.5 software). (**b**) Representative image of the 3D-printed specimen discs.

The samples’ 3D disk-shaped design, with a diameter of 12 mm and a height of 2 mm, was created using three-dimensional design software (Meshmixer v3.5, Autodesk Inc., San Francisco, CA, USA) and saved as an .stl file (Figure 1a). The .stl file was imported to a Digital Light Processing (DLP) 3D-printer (xPRINT, xDEPOT GmbH, Dachau, Germany) to produce 132 samples (Figure 1b).

Based on previous estimates, the sample size was calculated to achieve 80% statistical power at an α level of 0.05 and an effect size of 0.8 (G*Power software, version 3.1.9.4, Heinrich-Heine-Universität Düsseldorf, Düsseldorf, Germany). Therefore, a minimum of 14 samples was needed for a 30/60 min comparison of the tested materials.

Two denture base materials, V and F, were tested under two post-polymerization conditions (30 and 60 min), creating four groups: V30, V60, F30, and F60. For the direct testing, 9 discs per experimental group were used for the MTT, LDH, and NO tests, corresponding to 3 independent experiments with 3 discs per group. In addition, 3 discs per group were used for Live/Dead staining and 9 discs per group for caspase-3/7 analysis, resulting in a total of 21 discs per experimental group. For the indirect evaluation, 9 discs per experimental group were used for the MTT, LDH, and NO tests, and 3 discs per group were used for Holomonitor analysis, resulting in a total of 12 discs per experimental group and 48 discs for the indirect study.

The printing settings were set according to the manufacturer’s recommendation to 0-degree printing orientation, medium supports, a 100 mm layer height, and anti-aliasing level 4. After the printing process, post-processing included two cycles of rinsing for 3 min and 2 min in a 99% isopropyl alcohol bath using an ultrasonic wash station (Cavitek, Allendale Group Ltd., Hertfordshire, UK). After each rinsing cycle, the isopropyl solution was refreshed. At the end of the rinsing step, the disks were air-dried at room temperature using a compressed air duster and then exposed to two different polymerization procedures: half of the disks were polymerized for 30 min (F30 and V30). The remaining half (66) were cured for 60 min in a UV curing equipment (F60 and V60) (Form Cure L V1, Formlabs, Somerville, MA, USA), set with a 405 nm LED light source with an irradiance of 14.5 mW/cm^2^.

#### 3.1.3. Analysis of the Samples

Regarding the steps of analysis of surface characteristics and elemental composition and Si distribution, a scanning electron microscope (SEM, Hitachi TM3030PLUS Tabletop, Hitachi High-Tech Corporation, Tokyo, Japan) equipped with a system consisting of an energy-dispersive X-ray spectrometer (EDS, QUANTAX 70, Bruker Corporation, Billerica, MA, USA) with an integrated detector (XFlash 430H, Bruker Corporation, Billerica, MA, USA) was used. For the EDS measurements, the system was calibrated using the manufacturer’s standards prior to optimizing detection of light elements such as nitrogen, carbon, and oxygen, which are common in polymer-based dental resins. The EDS analysis was performed according to the protocol outlined in BS EN ISO 20795-1:2013, under 10 kV [23]. Samples were analyzed at different magnifications (×500, ×2000, ×5000, ×10,000, and ×20,000) to detect surface characteristics that may influence further analyses.

### 3.2. Cell Culture Preparation

The commercially available human gingival fibroblasts (hGF) cell line (CLS Cell Lines Service GmbH, Eppelheim, Germany; catalog number 300703) was used for this study. We mention that all procedures were conducted in accordance with institutional guidelines for the use of biological materials. The cells were cultivated in Dulbecco’s Modified Eagle’s medium (DMEM)/F12 with 10% fetal bovine serum and 1% antibiotic at a controlled temperature of 37 °C and 5% CO_2_ concentration. These were placed in 24-well plates at a density of 4 × 10^4^ cells per well and allowed to attach overnight, ensuring uniform seeding in 1 mL of culture medium per well. Afterward, the hGF cells were incubated with the samples for 24 h. Prior to testing, the samples were sterilized, followed by the protocol: 30 min immersion in 70% ethanol, air-dried, 30 min UV exposure on each side, and located centrally in each well plate to ensure a reliable specimen-to-well configuration. The control group consisted of cells that were not exposed to the material.

### 3.3. Assessment

All biological tests were performed in three independent experiments, each conducted under identical conditions and including triplicate technical measurements. Performing at least three replicates is widely accepted as the minimum number, according to ISO-10993-5-2009 standards: Biological evaluation of medical devices–Part 5: Tests for in vitro cytotoxicity [24]. For each experiment, three independent measurements were recorded (R1–R3), and the mean value was reported as the average (AVG). We conducted both direct and indirect assessments. In the direct study, we initially placed the cells, then added the material on top. Afterward, we removed the material and examined the cells under a microscope. The control group (C) consisted of cells not exposed to the discs. To validate the results from the direct study, we also performed indirect assessments, in which the medium was first incubated with the tested resins, then placed over the cells, which were examined under a microscope.

In the direct study, we evaluated: cell viability, nitric oxide production, cell membrane integrity, live/dead, and caspase-3/7 activity. In contrast, in the indirect study, assessments were conducted on cell viability, nitric oxide production, cell membrane integrity, and time-lapse live-cell activity.

#### 3.3.1. Cell Viability Assay (MTT Assay)

To assess cellular proliferation and cytotoxicity, a colorimetric method was performed using a solution of 3-(4,5-dimethylthiazol-2-yl)-2,5-diphenyltetrazolium bromide (Sigma-Aldrich, Darmstadt, Germany). The mechanism of this test is based on the enzymatic reduction of yellow tetrazolium salt (MTT) to insoluble purple formazan crystals by metabolically active cells. Thus, a working solution of MTT at 1 mg/mL was prepared. In each well, the cells were incubated with MTT reagent and culture medium at a 1:10 ratio (1 part reagent to 10 parts culture medium) for 2 h. After incubation, 400 µL of isopropanol was added to each well to solubilize the formed formazan crystals. Absorbance was measured at 590 nm and 630 nm using a FLUOstar^®^ Omega multi-mode microplate reader (BMG LABTECH, Ortenberg, Germany. The resulting absorbance values were correlated with the number of metabolically active cells present in the culture.

#### 3.3.2. Griess Assay (NO-Nitric Oxide Production Assay)

Nitric oxide (NO) levels were determined by measuring nitrite concentrations in the cell culture supernatants with the Nitric Oxide Assay Kit (Sigma-Aldrich, Burlington, MA, USA, Catalog number: MAK454). Culture media samples were collected after 24 h of incubation to assess changes in NO levels, which are known to increase during inflammation and oxidative stress. After 60 min of reaction, the absorbance was measured spectrophotometrically. Optical density readings were obtained at 540 nm using FLUOstar^®^ Omega (BMG LABTECH, Ortenberg, Germany).

#### 3.3.3. Lactate Dehydrogenase (LDH) Assay (Cell Membrane Integrity/Necrosis Assay)

The integrity of the cell membrane was evaluated by quantifying extracellular LDH released into culture media collected after 24 h of exposure to the samples. LDH activity was determined using the commercial LDH Cytotoxicity Assay kit (Sigma Aldrich Corporation, Burlington, MA, USA, REF: MAK006), according to the manufacturer’s instructions. The reaction was performed using a 1:1 ratio of culture medium to reagent. After a 25 min incubation in the dark, the absorbance was measured at 450 nm using FLUOstar^®^ Omega (BMG LABTECH, Ortenberg, Germany).

#### 3.3.4. Live/Dead Assays

Human gingival fibroblasts were seeded at a density of 2 × 10^4^ cells per well in a 24-well plate and left to adhere overnight. Subsequently, the cells were incubated for 24 h at 37 °C and 5% CO_2_ in the presence of the samples. Cell viability was then evaluated by applying the Viability/Cytotoxicity Assay Kit for animal live and dead cells (Biotium Inc., Fremont, CA, USA) for 30 min, following the guidelines provided by the manufacturer. The resulting fluorescence signals were captured through an inverted fluorescence microscope (IM-3LD4D, OPTIKA S.R.L., Ponteranica, Italy), and all images were subjected to further analysis using ImageJ software (version 1.54, National Institutes of Health, Bethesda, MD, USA).

#### 3.3.5. Caspase-3/7 Assays (Apoptosis Assay)

Caspase-3/7 activity, as a marker of apoptosis, was evaluated using a 5 µM solution of BioTracker NucView 488 Green Caspase-3 dye (Sigma-Aldrich, St. Louis, MO, USA), according to the manufacturer’s instructions. After hGF were exposed to the tested resins for 24 h, they were incubated with the dye for 30 min at 37 °C and protected from light. Intracellular caspase-3/7 activity was then measured using a microplate reader with excitation at 488 nm and emission at 520 nm. Fluorescent nuclei were visualized using an inverted fluorescence microscope (IM-3LD4D, OPTIKA S.R.L., Ponteranica, Italy), and apoptotic caspase-3–positive cells were quantified using ImageJ software (version 1.54, National Institutes of Health, Bethesda, MD, USA).

#### 3.3.6. Time-Lapse Live-Cell Imaging Analysis

For assessing cell migration and proliferation, the Holomonitor M4 digital holographic microscope (Phase Holographic Imaging PHI AB, Lund, Sweden) was used. Cells were seeded into 96-well plates and cultured in medium previously incubated with the tested resins. A HoloLid (Phase Holographic Imaging PHI AB, Lund, Sweden) was placed over the plate to enhance imaging quality and minimize evaporation. Time-lapse imaging was conducted every hour over a 24 h period. During the experiment, conditions were maintained inside the Holomonitor system in a standard humidified incubator at 37 °C with 5% CO_2_.

### 3.4. Statistical Analysis

Statistical analysis was performed using OriginPro software (v10.3, OriginLab Corporation, MA, USA). The data obtained from the test and control samples were statistically analyzed using Microsoft Excel (Microsoft Corp., WA, USA): mean, ratio, standard deviation, and *t*-test function. To compare groups statistically, one-way analysis of variance (ANOVA) was performed, with Tukey’s post hoc test used to identify specific differences between groups. Results were considered statistically significant at *p* ≤ 0.05.

## 4. Results

### 4.1. Elemental Analysis

**Figure 2:**
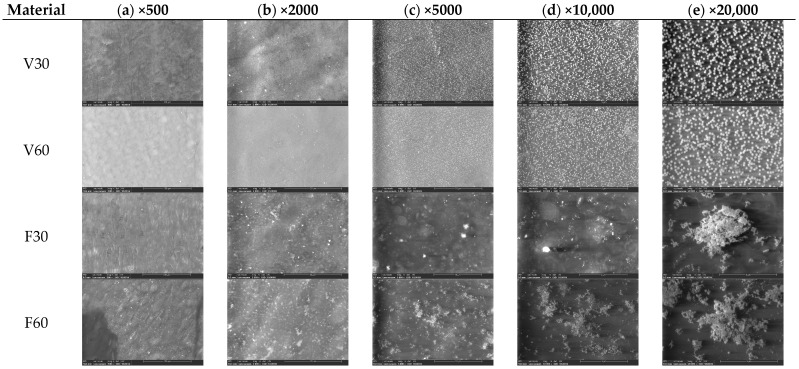
SEM images of 3D-printed specimens with different post-processing periods (magnification: (**a**) ×500; (**b**) ×2000; (**c**) ×5000; (**d**) ×10,000; (**e**) ×20,000).

The surface morphology of the 3D-printed denture base specimens was analyzed by scanning electron microscopy (SEM) at multiple magnifications (500×, 2000×, 5000×, 10,000×, and 20,000×) (Figure 2) to identify surface characteristics that may influence the cytocompatibility of the material. Variations in surface topography and particle-like structures may reflect differences in polymerization and post-processing efficiency.

**Figure 3:**
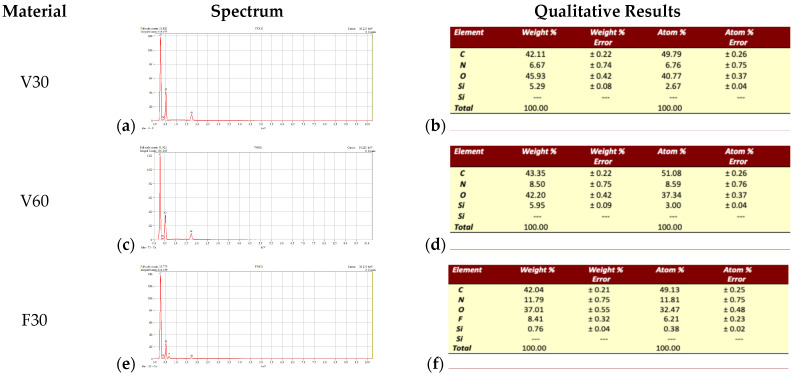

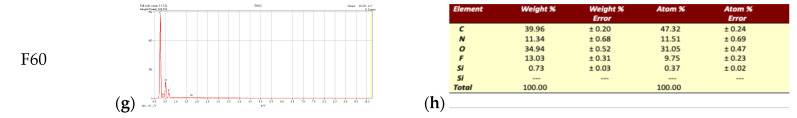
Energy-Dispersive X-ray Spectroscopy (EDS) Analysis: (**a**) EDS chart analysis of V samples postprocessed for 30 min; (**b**) EDS quantitative analysis of V samples postprocessed for 30 min; (**c**) EDS chart analysis of V samples postprocessed for 60 min; (**d**) EDS quantitative analysis of V samples postprocessed for 60 min; (**e**) EDS chart analysis of F samples postprocessed for 30 min; (**f**) EDS quantitative analysis of F samples postprocessed for 30 min; (**g**) EDS chart analysis of F samples postprocessed for 60 min; (**h**) EDS quantitative analysis of F samples postprocessed for 60 min.

It can be observed that the heterogeneous structure of material surfaces is evident only at magnifications greater than ×5000. Although the chemical compositions of the two materials are relatively similar (Figure 3), the polymeric surface structure is different. On different magnifications, starting with ×5000, the F material on both types of samples (F30 and F60) is present on the surface chains of not completely linked polymers. Unlike F material, the V materials are much clearer and more organized on the surface. At magnifications above ×10,000, SEM images illustrate more uniform surfaces for the V-Print dentbase samples, while for the FotoDent Denture specimens surface showed irregular polymer chains with visible clusters of silica. This heterogeneity of F surfaces might induce the retention of oxygen-inhibited layers, trap oligomers, and expose fillers.

### 4.2. Cell Viability Analysis (MTT Assay)

In the direct assessment (Table 2), the cell viability analysis showed that V30 reduced viability by 35% compared to the control, while V60 reduced viability by only 23%. In contrast, FotoDent Denture groups demonstrated a more pronounced reduction in viability, with F30 decreasing metabolic activity to approximately 42% of the control and F60 producing a similar value of 44%. Regarding indirect evaluation (Table 3), cell viability was reduced across all groups compared to the control. V30 and V60 showed approximately 59% and 53% viability, respectively, while FotoDent Denture increased from about 44% (F30) to 50% (F60).

**Figure 4:**
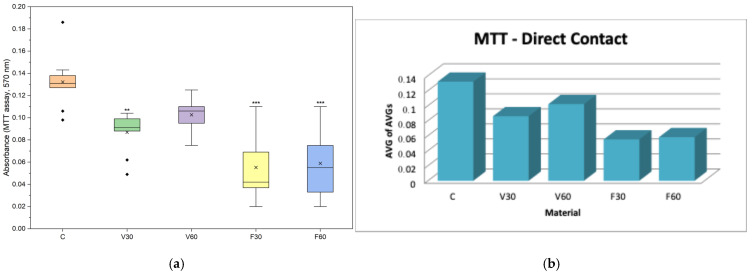
(**a**) Boxplot of MTT assay absorbance (570 nm) after 24 h direct contact of human gingival fibroblasts with the tested denture base materials (*n* = 9). ** *p* < 0.05, *** *p* < 0.005. (**b**) Mean MTT assay absorbance values (570 nm) of human gingival fibroblasts after 24 h of direct contact with the tested denture base materials.

In Figure 4a, data are presented as boxplots (interquartile range, median, mean, minimum). Statistical analysis was performed using one-way ANOVA followed by Tukey’s post hoc test. One-way ANOVA revealed significant differences among groups (F = 13.40, *p* < 0.0001). Tukey’s post hoc test showed a significant reduction in cell viability for V30 (*p* = 0.006) and a highly significant decrease for F30 and F60 (*p* < 0.0001) compared with the control group, while no significant difference was observed for V60 (*p* = 0.138). In Figure 4b, data are presented as bars representing the average of the mean values obtained from three independent experiments.

**Figure 5:**
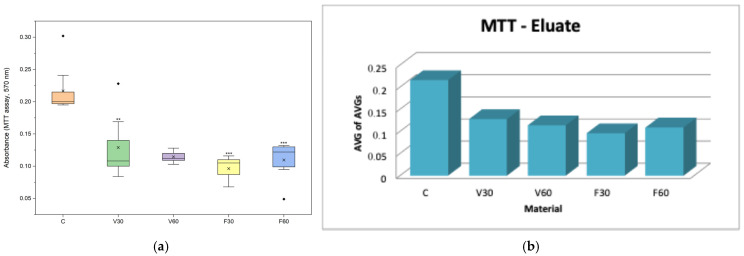
(**a**) Boxplot of MTT assay absorbance (570 nm) after 24 h of cell exposure to material eluates (*n* = 9). ** *p* < 0.05, *** *p* < 0.005. (**b**) Mean MTT assay absorbance values (570 nm) of human gingival fibroblasts after 24 h of exposure to material eluates.

In Figure 5a, data are presented as boxplots (interquartile range, median, mean, minimum). Statistical analysis was performed using one-way ANOVA followed by Tukey’s post hoc test. Exposure to V30, F30, and F60 eluates resulted in significantly lower MTT absorbance values compared to the control, indicating reduced cell viability, while V60 induced less significant cytotoxic effect. In Figure 5b, data are presented as bars representing the average of the mean values obtained from three independent experiments.

### 4.3. Griess Assay (NO-Nitric Oxide Production Assay)

In both assessments (Table 4, Table 5), NO levels, all groups showed relatively uniform changes, suggesting that the tested materials did not trigger detectable nitrosative-induced inflammatory reactions at different polymerization times under direct exposure conditions.

**Figure 6:**
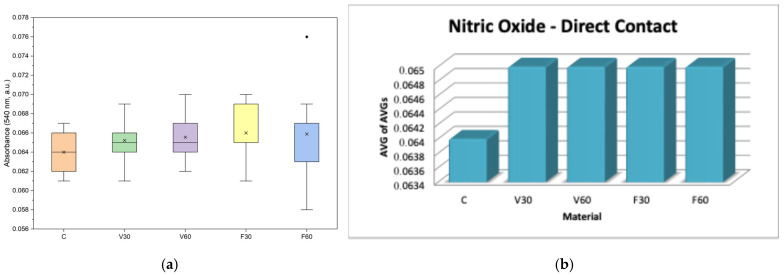
(**a**) Boxplot of nitric oxide (NO) production, expressed as absorbance at 540 nm, in human gingival fibroblasts after 24 h of direct contact with the tested denture base materials (*n* = 9). (**b**) Mean Griess assay absorbance values (540 nm) of human gingival fibroblasts after 24 h of direct contact with the tested denture base materials.

In Figure 6a, data are presented as boxplots (interquartile range, median, mean, minimum). One-way ANOVA revealed no statistically significant differences in nitric oxide levels among the experimental groups and the control (F = 0.52, *p* = 0.72). Post hoc analysis using Tukey’s multiple comparison test confirmed that none of the tested materials (V30, V60, F30, F60) induced a significant change in no production compared with the control group (*p* > 0.05 for all comparisons). These findings indicate that exposure to material eluates did not trigger an inflammatory response in human gingival fibroblasts, as reflected by stable nitric oxide levels across all experimental conditions. In Figure 6b, data are presented as bars representing the average of the mean values obtained from three independent experiments.

**Figure 7:**
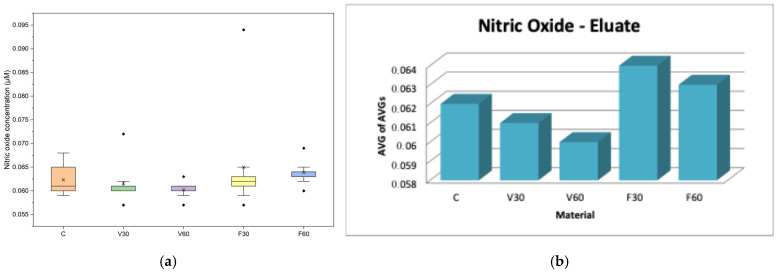
(**a**) Boxplot of nitric oxide (NO) production in human gingival fibroblasts after 24 h exposure to material eluates (*n* = 9). (**b**) Mean Griess assay absorbance values of human gingival fibroblasts after 24 h of exposure to material eluates.

In Figure 7a, data are presented as boxplots (interquartile range, median, mean, minimum). One-way ANOVA showed no statistically significant differences in nitric oxide levels among the tested materials after 24 h of exposure (*p* > 0.05). Post hoc Tukey analysis confirmed the absence of significant pairwise differences, indicating that none of the tested materials induced a significant change in NO production compared to the control. In Figure 7b, data are presented as bars representing the average of the mean values obtained from three independent experiments.

### 4.4. Lactate Dehydrogenase (LDH) Assay (Cell Membrane Integrity/Necrosis Assay)

LDH activity analysis in the direct evaluation showed clear differences between the two materials across all tested groups (Table 6). V30 showed a 5% increase in LDH release compared to the control, whereas V60 showed only a 1% difference; neither reached statistical significance (*p* > 0.05). For FotoDent Denture, F30 and F60 showed statistically significant LDH increases of 13% and 18%, respectively (*p* ≤ 0.05). The eluate groups (Table 7) showed that V30 recorded a 9% increase compared to the control, while V60 showed a 15% reduction. For FotoDent Denture, F30 showed LDH values 18% below the control, whereas F60 exhibited a 60% increase, reflecting substantial membrane damage with extended polymerization.

**Figure 8:**
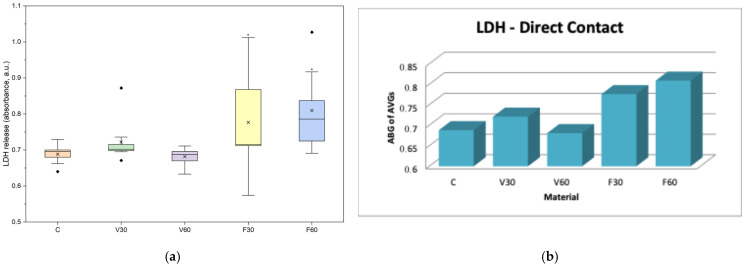
(**a**) Boxplot of LDH release after 24 h direct contact of human gingival fibroblasts with the tested denture base materials (*n* = 9). * *p* < 0.05. (**b**) Mean LDH assay of human gingival fibroblasts after 24 h of direct contact with the tested denture base materials.

In Figure 8a, data are presented as boxplots (interquartile range, median, mean, minimum). One-way ANOVA revealed a statistically significant difference in LDH release among the tested groups after 24 h of direct contact (F = 4.13, *p* = 0.0068). Post hoc Tukey analysis showed significantly higher LDH release for F30 and F60 compared to the control group (*p* < 0.05), while no significant differences were observed between the control and V30 or V60 groups. In Figure 8b, data are presented as bars representing the average of the mean values obtained from three independent experiments.

**Figure 9:**
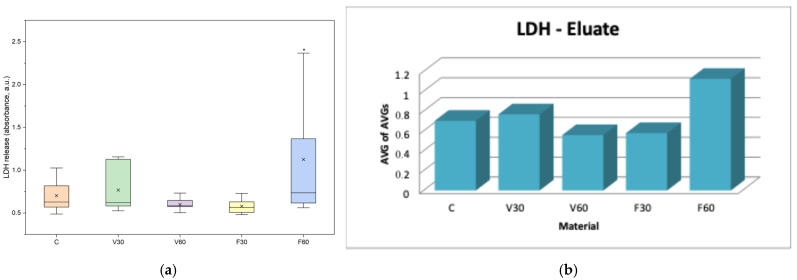
(**a**) Boxplot of LDH release in human gingival fibroblasts after 24 h exposure to material eluates (*n* = 9). * *p* < 0.05. (**b**) Mean LDH assay absorbance values of human gingival fibroblasts after 24 h of exposure to material eluates.

In Figure 9a, data are presented as boxplots (interquartile range, median, mean, minimum). One-way ANOVA revealed significant differences in LDH release among groups after 24 h of exposure (F = 3.28, *p* = 0.020). Tukey’s post hoc analysis showed significantly higher LDH release for F60 compared to the control, V60, and F30 groups (*p* < 0.05), while no other significant differences were observed. In Figure 9b, data are presented as bars representing the average of the mean values obtained from three independent experiments.

### 4.5. Caspase-3/7 Assays (Apoptosis Assay)

All groups exhibited elevated caspase-3/7 activity relative to the control (Table 8). In the V group, V30 and V60 recorded increases of 11% and 8%, respectively. A similar pattern was observed for FotoDent Denture, where F30 showed a 20% increase, while F60 decreasing by 12%. In both materials, longer polymerization time was linked to lower apoptotic signaling, yet caspase activity remained above control levels across all conditions.

**Figure 10:**
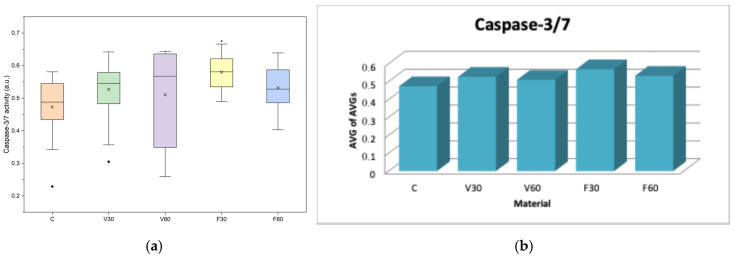
(**a**) Boxplot of caspase-3/7 activity in human gingival fibroblasts after 24 h of direct contact with the tested denture base materials (*n* = 18). * *p* < 0.05. (**b**) Mean caspase-3/7 activity of human gingival fibroblasts after 24 h of direct contact with the tested denture base materials.

In Figure 10a, data are presented as boxplots (interquartile range, median, mean, minimum). One-way ANOVA revealed significant differences in caspase-3/7 activity among the tested groups after 24 h of direct contact (F = 2.99, *p* = 0.023). Post hoc Tukey analysis indicated a significantly higher caspase-3/7 activity for F30 compared to the control group (*p* < 0.05), while no other pairwise comparisons were statistically significant. In Figure 10b, data are presented as bars representing the average of the mean values obtained from three independent experiments.

### 4.6. Statistical Analysis

Dendrograms indicate hierarchical clustering based on similarity of response profiles. The F30 and F60 clusters reflect reduced cell viability and increased apoptotic and cytotoxic markers compared to the control, whereas the V60 cluster is closer to the control, indicating a more favorable biological response. V30 exhibited an intermediate profile, highlighting material- and post-curing-time-dependent effects.

**Figure 11:**
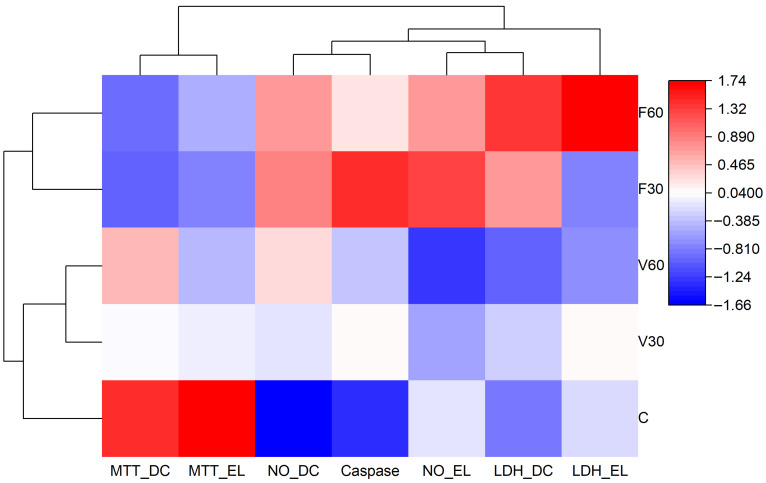
Hierarchically clustered heatmap summarizing the biological responses of human gingival fibroblasts to the tested denture base materials.

In Figure 11, data are presented as a hierarchically clustered heatmap. Rows represent experimental groups (C, V30, V60, F30, F60). Columns represent the assessment parameters: MTT—direct contact (MTT_DC), MTT—eluates (MTT_EL), nitric oxide—direct contact (NO_DC), caspase-3/7 activity, nitric oxide—eluates (NO_EL), lactate dehydrogenase—direct contact (LDH_DC), and lactate dehydrogenase—eluates (LDH_EL). Data were normalized and color-coded according to relative intensity (blue: lower values; red: higher values).

### 4.7. Live/Dead Assays

**Figure 12:**
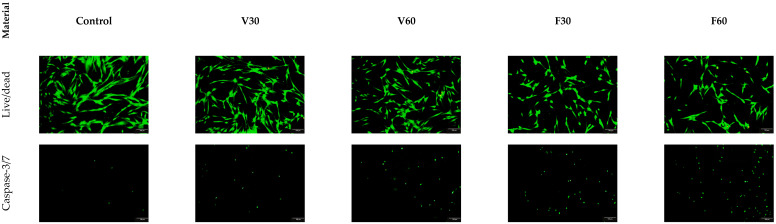
Representative fluorescent microscopy images of hGF after 24 h of direct exposure to the sample. Live/Dead staining (**upper row**) shows viable cells in green, while caspase-3/7 staining (**lower row**) highlights apoptotic nuclei in green.

The Live/Dead staining demonstrated that the majority of cells exhibited green fluorescence, indicating that membrane integrity was mostly preserved. Cells generally maintained a spindle-like fibroblastic morphology; however, differences in cell density and spreading were noticeable among the experimental groups compared to the control. Caspase-3/7 staining revealed scattered green fluorescent nuclei across all specimen groups, confirming the presence of apoptotic cells. The fluorescence signal appeared localized rather than general, with most cells absent detectable caspase signal, suggesting that apoptosis was present but not dominant under these exposure parameters (Figure 12).

### 4.8. Time-Lapse Live-Cell Imaging Analysis

Holomonitor time-lapse live-cell imaging was used to evaluate human gingival fibroblast morphology and density across all experimental groups at baseline (0 h) and following 24 h of exposure, providing a real-time visual assessment of cell behavior in response to each material.

**Figure 13:**
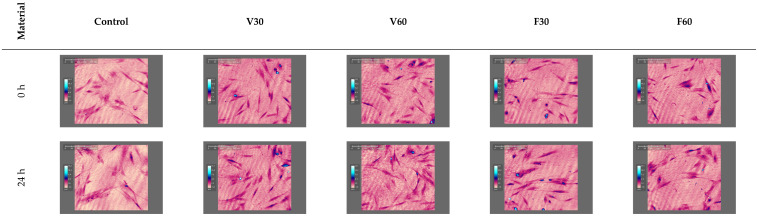
Holomonitor representative time-lapse live-cell imaging captured at 0 h and after 24 h.

In Figure 13, the record at 0 h and 24 h of hGF exposed to the tested resins is shown on the holomonitor. Although minor variations in cell density and distribution can be noticed between experimental groups, no significant structural abnormalities are visually apparent.

## 5. Discussion

In recent years, significant research attention has been focused on evaluating the biological behavior of light-curable denture base resins used in additive manufacturing [9,25,26,27,28,29,30,31]. Even with improvements in resin composition, there are still concerns about their cytotoxic potential, which is often linked to incomplete polymerization and residual substances. Therefore, we proposed to investigate the influence of post-curing conditions on the surface characteristics and cytotoxicity of a 3D-printed denture base polymer.

The presence of residual components after polymerization, such as monomer, additive, or photoinitiator, could lead to an initial depletion of the cell’s glutathione levels and disruption of its normal redox status. It is well known that this results in the formation of reactive oxygen species (ROS), which leads to oxidative stress [16,32,33]. Oxidative stress has been shown to inhibit cellular metabolic processes, to activate apoptosis-related pathways, and, depending upon the concentration of the toxicants near the surface of the cells, to disrupt membrane integrity [6,34].

The effects of the tested denture base resins on cellular behavior were initially assessed through direct tests, including cell viability, nitric oxide production, cell membrane integrity, live/dead and caspase-3/7 activity. For assessing cellular viability after 24 h of evaluation, all tested denture base materials showed reduced MTT values compared with the control. V30 reduced viability by 35% compared to the control, while V60 reduced viability by only 23%, indicating that a longer polymerization time was associated with higher metabolic activity for the V-Print dentbase resin. Similar results were found in many of the recently published studies on additively manufactured denture base resins. In these studies, increasing post-curing time increased the degree of conversion and reduced the amount of unreacted monomer released into the medium, leading to improved cell viability [27,35]. In contrast, FotoDent Denture groups demonstrated a more pronounced reduction in viability, with F30 decreasing metabolic activity to approximately 42% of the control and F60 producing a similar value of 44%, suggesting that the biological reaction can vary based upon the type of resin used and the curing efficiencies of the resin, as has been reported for other printable denture base materials [36].

Regarding NO levels, all groups showed relatively uniform changes, suggesting that the tested materials did not trigger detectable nitrosative-induced inflammatory reactions at different polymerization times under direct exposure conditions. Previous studies have found that the cytotoxic effects of denture base polymers are primarily due to the release of unreacted monomers, oligomers, or photoinitiator and thus typically do not significantly increase in vitro nitric oxide production even after short-term exposure [37]. Therefore, the observed metabolic viability and membrane effects were most likely due to direct cellular toxicity rather than to an NO-induced inflammatory response. On the other hand, cell membrane integrity, measured through LDH activity in V30, increased by 5% compared to the control, while V60 showed values close to the control (99%), indicating no statistical significance and suggesting that prolonged polymerization was associated with reduced membrane disturbance for V-Print dentbase. In contrast, FotoDent Denture showed higher LDH levels than the control, with increases of 13% for F30 and 18% for F60. Thus, while prolonged polymerization was associated with decreased LDH release in the V group, the opposite trend was observed for Fotodent in direct contact. Evaluating the literature, previous studies have shown how different formulations for both acrylic and photopolymer dental materials can affect LDH release regardless of low metabolic activity due to the residual monomer and/or the degree of incomplete curing, which could modify cell membrane permeability in addition to causing an initial cytotoxic effect [38,39,40]. These findings support the premise that while there is a trend toward reduced biological impact with better polymerization, ultimately the final cellular reaction is still influenced by both the composition of the material as well as curing parameters which result in varying degrees of membrane damage among denture base polymers.

Considering apoptotic activity assessed by caspase-3/7, all materials showed increased activity compared with the control. V30 induced an increase with 11%, while V60 with 8%. Similarly, F30 increased caspase activity with 20%, whereas F60 decreased with 12%. For both resins, extending polymerization time was associated with reduced apoptotic signaling, although caspase activity remained above control levels. Kollmuss et al. [6] demonstrated that both the release of unreacted monomers and partial curing of dental resins activate apoptosis in cells such as fibroblasts. The authors found that complete curing resulted in a decrease in cellular stress; however, it was not eliminated. In addition, Chang et al. [41] demonstrated that the degree of cure of photopolymerizable dental resins had an impact on biological responses. Specifically, inadequate curing of these resins led to higher levels of cytotoxicity and activation of the processes by which cell death is induced. According to our results, the decrease in MTT levels and the increase in caspase-3/7 activity, and a non-significant alteration of LDH level in the V group, are most consistent with a reduced overall apoptosis-induced cell stress response rather than a necrosis process. The combination of decreased MTT with high levels of LDH in the F-exposed cells suggests a mixed type of cytotoxicity—membrane damage combined with apoptosis. The significantly increased caspase 3/7 activity in the F30-exposed cells indicated that apoptosis contributed to the loss of viability, as illustrated by the MTT results.

To double the direct assessments, indirect evaluation was carried out, including cell viability, nitric oxide production, cell membrane integrity, and time-lapse live-cell activity.

For the MTT, viability was reduced for all groups compared with the control. V30 and V60 resulted in approximately 59% and 53% viability, respectively, indicating a moderate decrease with extended polymerization. In contrast, FotoDent Denture increased from approximately 44% (F30) to 50% (F60), suggesting an improvement in metabolic activity with longer curing. Regarding cell membrane integrity, LDH levels demonstrated a reduced increase in activity for V30 (9% compared to the control), while for V60, a 15% reduction, supporting a lower membrane-damaging potential of eluates after a longer polymerization period for the V group. For the F group, F30 showed LDH values below the control (82%), whereas F60 showed a marked 60% increase, indicating substantial membrane damage associated with eluates from this condition. Taken together, these findings support the direct observations by confirming that leachable components contribute to reduced metabolic activity in both materials. Regarding NO levels, there were no significant differences between the groups, indicating that nitric oxide production does not change the eluates’ parameters.

Synthesizing our findings, V-Print dentbase indicates that a longer polymerization time was associated with improved biological behavior, as follows: higher MTT values under direct contact, LDH values closer to the control, and reduced caspase-3/7 activity. In the indirect assessment, the marked decrease in LDH for V60 further supports reduced membrane stress from eluates, sustaining the direction of the previous results. For FotoDent Denture, the findings of direct and indirect studies suggest a variable response to prolonged polymerization. In contact between human gingival fibroblasts with disks, MTT values remained similarly reduced for both polymerization times, while caspase-3/7 activity decreased, suggesting reduced apoptotic signaling. However, LDH increases under direct conditions, indicating greater membrane damage associated with necrotic processes or late-stage apoptosis. In the indirect assessment, the improvement in F60 viability was accompanied by a marked increase in LDH, indicating substantial membrane damage. Hence, while extended polymerization reduced caspase-3/7 activity in FotoDent Denture, LDH levels differed across exposure times. This suggests that the biological response of the F group may depend on resin-specific factors, such as complex acrylate structures, photoinitiator behavior, or post-processing sensitivity, rather than on polymerization time alone. Such variability has been reported in the literature, as some studies confirm improved cytocompatibility with extended curing, while others fail to detect uniform improvements beyond manufacturer protocols [14,15,42,43].

Notably, when comparing the assessment methods, the direct contact approach usually caused more significant cellular changes than the eluate-based evaluation. This conclusion is expected because fibroblasts are exposed to the immediately tested 3D-printed denture base material interface. Under these conditions, cells are influenced not only by saliva-soluble substances released from the resin, such as residual monomers, but also by 3D printing parameters, including layer thickness, build orientation, and surface roughness [44,45,46], which can cause surface irregularities or oxygen-inhibited superficial layers that can retain unreacted substances and increase the local biological response of oral tissue. This suggests the importance of understanding and following the printing parameters. In contrast, indirect eluate testing evaluates the contribution of diffusible substances, which are diluted in clinical conditions. Thus, when assessing denture base biocompatibility, it is relevant to evaluate both exposure scenarios.

Regarding the SEM images (Figure 2), differences in surface morphology among the denture base resins were observed. For the V group (V30 and V60), the surfaces appeared homogeneous at lower magnifications. V60 presented a more uniform and smoother microstructure compared with V30, suggesting that extended post-curing may enhance polymer structure. These microscopy images are consistent with the biological findings, in which V60 exhibited a more favorable cellular response under prolonged polymerization conditions. On the other hand, the F specimens (F30 and F60) displayed markedly different surface profiles. Even at lower magnifications, surfaces appeared more heterogeneous, with irregularities. At higher magnifications, both F30 and especially F60 demonstrated pronounced clustered structures and rough surfaces, which may correspond to inorganic nanoparticles of silica compounds detected over EDS investigation (Figure 3). The presence of silicon-based fillers or siloxane-related additives in FotoDent may contribute to increased surface roughness and microstructural heterogeneity, potentially affecting the interaction at the edge between human gingival fibroblasts and material. Such an irregular surface can retain residual resin components. The impact of surface morphology on 3D-printed denture bases is also relevant to post-processing. The results from the above-mentioned authors, Li et al. [43] and Dai et al. [47], show that surface properties can be significantly influenced by post-processing and therefore can affect cytotoxic effects. Additionally, several studies on denture base resin have demonstrated that a smooth and regular surface morphology is related to better surface properties, while an uneven and irregular surface may lead to higher material–cell interactions and the greater retention of biofilm or other substances on the surface [25,48]. Therefore, our findings may explain the larger differences in direct MTT and LDH results for F samples compared to eluate effects, because surface features generate concentration points of interface challenge that are less apparent in eluates.

Another important aspect that may explain the different biological responses observed between the tested materials is their individual chemical formulation. The V-Print dentbase resin is primarily composed of aliphatic urethane dimethacrylate (UDMA) as the main monomer, together with smaller quantities of triethylene glycol dimethacrylate (TEGDMA) and the photoinitiator bisacylphosphine oxide (BAPO). These compounds belong to the class of urethane-based dimethacrylates, which polymerize into relatively stable cross-linked networks but may still release residual dimethacrylate monomers when conversion is incomplete [6,49]. In contrast, FotoDent Denture resin presents a more complex composition, joining multifunctional acrylate and methacrylate oligomers, including polyether diacrylate structures, a urethane-based dimethacrylate component, and particularly aliphatic urethane triacrylate, together with the photoinitiator diphenyl(2,4,6-trimethylbenzoyl)phosphine oxide (TPO). The triacrylate monomers are known to form denser and heterogeneous cross-linked polymer networks and may maintain unreacted oligomeric fractions of residual monomers, which may subsequently elute in biological conditions [4,11,50].

BAPO and TPO, as phosphine oxide initiators, are part of the Norrish type-I photoinitiators category and are used in vat-photopolymerized dental materials due to their high reactivity and efficient radical generation under UV light exposure in the absorption spectrum of 365–416 nm [6,51]. Due to differences in their absorption spectra and potential leaching behavior, they may influence resin-specific cytotoxicity if residual fractions remain unreacted [6,10,13,52]. In the present study, post-polymerization was performed using a Form Cure unit with a 405 nm LED light source [53], which falls within the absorption spectrum of the photoinitiators. However, differences in resin formulation may still interact with the rate and extent of polymerization under the same curing conditions. Van Landuyt et al. [54] reported that eluates from TPO-based systems may exhibit higher cytotoxicity despite lower monomer release, suggesting that photoinitiator-related leaching may contribute independently to cell aggression responses [52]. Therefore, differences in surface and monomer classes, as well as in photoinitiator formulations, may contribute to cytotoxicity patterns and explain the more consistent improvements observed for the V group compared with the F group.

Based on our results, the hypothesis was approved for the V group, extending polymerization time produced reliable benefit improvements, whereas the F group exhibited a more variable biological response, suggesting that the extension of time for post-curing may be correlated with resin chemistry.

Regarding limitations of the study, it should be mentioned that this research was designed under in vitro conditions, which are unable to replicate the mechanisms related to toxicity and inflammation from the oral cavity. Moreover, the choice of elution liquid was limited by the requirement that the extraction medium be compatible with the cells used in the in vitro testing. A further limitation of this study is that the specimens were exposed for only 24 h and were not subjected to artificial aging or cyclic fatigue. Since complete dentures are exposed to mastication, the quantity and quality of components leached into saliva may differ in vivo. Accordingly, it was useful to determine the degree of conversion of the tested resins to confirm that the leachable substances are due to residual monomer. Moreover, considering the different composition and optical properties of resins, such as opacity, it is important to account for light penetration and curing depth. Therefore, future studies should evaluate the chemical composition of vat-photopolymerized denture base resins, the monomer’s influence on the long-term biocompatibility of denture base resins and the potential influence of variations in cure depth on the cytotoxicity of the material.

## 6. Conclusions

In summary, based on the findings of this in vitro study, the following conclusions were drawn:

1. Post-curing duration influenced the viability and cell metabolic activity of the evaluated 3D-printed denture base resins. For the V group, prolonged polymerization was associated with higher viability, membrane integrity values closer to the control, and reduced apoptosis, indicating a more favorable cellular response compared to the F group.
2. Direct contact conditions induced more evident cellular alterations than eluate-based testing, supporting the idea that biological response is conditional on both residual 3D-printed denture base resin components and surface characteristics.

From a clinical perspective, these indicate that post-processing protocols should be carefully optimized for each resin system rather than applied uniformly, as incomplete or insufficient post-curing may increase the release of biologically active residual substances and affect interactions with oral tissues.

## Figures and Tables

**Table 1:** The composition and post-processing parameters of the two 3D-printed denture base resins included in the study.

3D-Printed Resin	Lot Number	Composition		Post-Processing	
		Substance	%	Cleaning	Post-Curing
FotoDent Denture, 385 nm, pink transparent	010058X0	Poly[oxy(methyl-1,2-ethanediyl)],.alpha.,.alpha’.-(2,2-dimethyl-1,3-propanediyl)bis[.omega.-[(1-oxo-2-propenyl)oxy]	≥25% <50%	Ultrasonic bath with isopropanol solution—preliminary cleaning (2 min) and final cleaning (2 min) with fresh isopropanol	UV-lightbox at 90 °C for 10 min
		7,7,9(7,9,9)-trimethyl-4,13-dioxo-3,14-dioxa-5,12-diazahexadecane-1,16-diylbismethacrylate	≥25% <50%		
		aliphatic urethane triacrylate	≥10 <25%		
		diphenyl(2,4,6-trimethylbenzoyl)phosphine oxide (TPO)	≥1% <3%		
		dichlorodimethylsilane	≥0.1% <2%		
V-Print dentbase	2404745	Aliphatic urethane dimethacrylate (UDMA)	>50% ≤75%	Ultrasonic bath with isopropanol solution—preliminary cleaning (3 min) and final cleaning (2 min) with fresh isopropanol	UV-lightbox at 90 °C for 30 min
		TEGDMA (triethylene glycol dimethacrylate)	>2.5% ≤10%		
		Phenylbis (2,4,6-trimethylbenzoyl)phosphine oxide (BAPO)	0.01% −1%		

**Table 2:** The results obtained from the MTT assay after 24 h of direct contact between human gingival fibroblasts and the tested denture base materials.

Material	Experiment 1				Experiment 2				Experiment 3			
	R1	R2	R3	AVG	R1	R2	R3	AVG	R1	R2	R3	AVG
C	0.138	0.186	0.132	0.152	0.143	0.129	0.131	0.134	0.098	0.106	0.127	0.110
V30	0.049	0.062	0.098	0.069	0.091	0.099	0.103	0.097	0.088	0.104	0.088	0.093
V60	0.104	0.11	0.106	0.106	0.095	0.11	0.117	0.107	0.082	0.075	0.125	0.094
F30	0.108	0.11	0.042	0.086	0.037	0.026	0.02	0.027	0.042	0.043	0.069	0.051
F60	0.106	0.11	0.02	0.078	0.023	0.05	0.075	0.049	0.058	0.055	0.033	0.048

**Table 3:** The results obtained from the MTT assay after 24 h of cell exposure to material eluates.

Material	Experiment 1				Experiment 2				Experiment 3			
	R1	R2	R3	AVG	R1	R2	R3	AVG	R1	R2	R3	AVG
C	0.215	0.206	0.241	0.220	0.2	0.195	0.197	0.197	0.199	0.195	0.302	0.232
V30	0.127	0.228	0.105	0.153	0.1	0.084	0.108	0.097	0.14	0.169	0.1	0.136
V60	0.122	0.109	0.103	0.111	0.128	0.112	0.12	0.12	0.112	0.116	0.109	0.112
F30	0.105	0.092	0.068	0.088	0.11	0.111	0.116	0.112	0.105	0.087	0.073	0.088
F60	0.122	0.13	0.13	0.127	0.049	0.095	0.102	0.082	0.132	0.128	0.099	0.119

**Table 4:** The results obtained from the Griess assay after 24 h of direct contact between human gingival fibroblasts and the tested denture base materials.

Material	Experiment 1				Experiment 2				Experiment 3			
	R1	R2	R3	AVG	R1	R2	R3	AVG	R1	R2	R3	AVG
C	0.066	0.062	0.061	0.063	0.067	0.064	0.064	0.065	0.063	0.062	0.067	0.064
V30	0.065	0.064	0.069	0.066	0.066	0.064	0.069	0.066	0.061	0.066	0.063	0.063
V60	0.067	0.07	0.064	0.067	0.068	0.067	0.065	0.066	0.065	0.062	0.062	0.063
F30	0.07	0.065	0.069	0.068	0.065	0.068	0.069	0.067	0.065	0.061	0.062	0.062
F60	0.065	0.069	0.067	0.067	0.076	0.067	0.067	0.07	0.063	0.061	0.058	0.060

**Table 5:** The results obtained from the Griess assay after 24 h of cell exposure to material eluates.

Material	Experiment 1				Experiment 2				Experiment 3			
	R1	R2	R3	AVG	R1	R2	R3	AVG	R1	R2	R3	AVG
C	0.059	0.059	0.06	0.059	0.065	0.067	0.06	0.064	0.062	0.068	0.061	0.063
V30	0.057	0.061	0.072	0.063	0.061	0.06	0.06	0.060	0.061	0.062	0.06	0.061
V60	0.059	0.061	0.061	0.060	0.061	0.06	0.06	0.060	0.06	0.057	0.063	0.06
F30	0.065	0.061	0.059	0.061	0.063	0.094	0.063	0.073	0.062	0.057	0.061	0.06
F60	0.062	0.065	0.069	0.065	0.064	0.06	0.064	0.062	0.063	0.064	0.064	0.063

**Table 6:** The results obtained from the LDH assay after 24 h of direct contact between human gingival fibroblasts and the tested denture base materials.

Material	Experiment 1				Experiment 2				Experiment 3			
	R1	R2	R3	AVG	R1	R2	R3	AVG	R1	R2	R3	AVG
C	0.699	0.68	0.696	0.691	0.7	0.705	0.729	0.711	0.685	0.662	0.64	0.662
V30	0.872	0.698	0.736	0.768	0.715	0.701	0.695	0.703	0.707	0.702	0.671	0.693
V60	0.707	0.693	0.673	0.691	0.67	0.687	0.633	0.663	0.666	0.711	0.696	0.691
F30	0.574	0.868	1,012	0.818	0.712	0.715	0.712	0.713	0.896	0.818	0.685	0.799
F60	0.72	1,027	0.917	0.888	0.786	0.691	0.725	0.734	0.837	0.75	0.835	0.807

**Table 7:** The results from the LDH assay after 24 h of cell exposure to material eluates.

Material	Experiment 1				Experiment 2				Experiment 3			
	R1	R2	R3	AVG	R1	R2	R3	AVG	R1	R2	R3	AVG
C	0.488	0.628	0.569	0.561	0.568	0.817	1.026	0.803	0.868	0.776	0.592	0.745
V30	0.525	0.56	0.582	0.555	0.583	0.622	0.621	0.608	1.156	1.14	1.128	1.141
V60	0.577	0.521	0.504	0.534	0.574	0.733	0.589	0.632	0.584	0.648	0.665	0.632
F30	0.48	0.507	0.49	0.492	0.567	0.554	0.73	0.617	0.607	0.63	0.647	0.628
F60	0.95	0.735	0.627	0.770	0.617	0.57	0.562	0.583	1.366	2.337	2.367	2.023

**Table 8:** Caspase-3/7 activity in human gingival fibroblasts (hGF) after 24 h direct contact with the tested 3D-printed denture base materials.

Material	Experiment 1							Experiment 2							Experiment 3						
	R1	R2	R3	R4	R5	R6	AVG	R7	R8	R9	R10	R11	R12	AVG	R13	R14	R15	R16	R17	R18	AVG
C	0.545	0.575	0.481	0.560	0.485	0.434	0.513	0.420	0.468	0.343	0.569	0.495	0.581	0.479	0.505	0.512	0.229	0.473	0.518	0.352	0.432
V30	0.357	0.305	0.436	0.526	0.483	0.641	0.458	0.642	0.633	0.632	0.418	0.544	0.538	0.568	0.508	0.569	0.547	0.551	0.579	0.576	0.555
V60	0.566	0.596	0.636	0.585	0.563	0.639	0.597	0.259	0.447	0.268	0.533	0.569	0.644	0.453	0.613	0.643	0.640	0.303	0.339	0.349	0.481
F30	0.535	0.548	0.557	0.602	0.577	0.586	0.568	0.647	0.531	0.621	0.585	0.532	0.534	0.575	0.466	0.490	0.536	0.635	0.610	0.652	0.565
F60	0.506	0.506	0.522	0.486	0.434	0.474	0.488	0.539	0.436	0.403	0.574	0.587	0.596	0.522	0.593	0.534	0.519	0.626	0.609	0.639	0.587

## Data Availability

The original contributions presented in the study are included in the article; further inquiries can be directed to the corresponding authors.

## Abbreviations

The following abbreviations are used in this manuscript:

3D-prining	Three-dimensional printing
AVG	Average
AVG of AVGs	Average of averages
BAPO	Phenylbis(2,4,6-trimethylbenzoyl)phosphine oxide
C	Control
CAM-CAM	Computer-aided design and computer-aided manufacturing
DLP	Digital light processing
DMEM	Dulbecco’s Modified Eagle Medium
EDS	Energy-Dispersive Spectroscopy
F	FotoDent Denture material
hGF	Human gingival fibroblasts
ISO	International Organization for Standardization
LDH	Lactate dehydrogenase
LED	Light-Emitting Diode
MTT	3-(4,5-Dimethylthiazol-2-yl)-2,5-diphenyltetrazolium bromide
NO	Nitric oxide
PMMA	Polymethyl methacrylate
SEM	Scanning electron microscopy
SLA	Stereolithography
STL	Standard tessellation language
TEGDMA	Triethylene glycol dimethacrylate
TPO	Diphenyl(2,4,6-trimethylbenzoyl)phosphine oxide
UDMA	Urethane dimethacrylate
UV	Ultraviolet
V	V-Print dentbase material
